# Continuous neural control of a bionic limb restores biomimetic gait after amputation

**DOI:** 10.1038/s41591-024-02994-9

**Published:** 2024-07-01

**Authors:** Hyungeun Song, Tsung-Han Hsieh, Seong Ho Yeon, Tony Shu, Michael Nawrot, Christian F. Landis, Gabriel N. Friedman, Erica A. Israel, Samantha Gutierrez-Arango, Matthew J. Carty, Lisa E. Freed, Hugh M. Herr

**Affiliations:** 1 K. Lisa Yang Center for Bionics, Massachusetts Institute of Technology, Cambridge, MA USA; 2 Harvard-MIT Division of Health Sciences and Technology, Massachusetts Institute of Technology, Cambridge, MA USA; 3 Media Arts and Sciences, Massachusetts Institute of Technology, Cambridge, MA USA; 4 Mechanical Engineering Department, Massachusetts Institute of Technology, Cambridge, MA USA; 5 Department of Neurosurgery, Massachusetts General Hospital and Harvard Medical School, Boston, MA USA; 6 Division of Plastic and Reconstructive Surgery, Brigham and Women’s Hospital, Boston, MA USA; 7 McGovern Institute, Massachusetts Institute of Technology, Cambridge, MA USA

**Keywords:** Biomedical engineering, Mechanical engineering, Medical research, Motor control

## Abstract

For centuries scientists and technologists have sought artificial leg replacements that fully capture the versatility of their intact biological counterparts. However, biological gait requires coordinated volitional and reflexive motor control by complex afferent and efferent neural interplay, making its neuroprosthetic emulation challenging after limb amputation. Here we hypothesize that continuous neural control of a bionic limb can restore biomimetic gait after below-knee amputation when residual muscle afferents are augmented. To test this hypothesis, we present a neuroprosthetic interface consisting of surgically connected, agonist–antagonist muscles including muscle-sensing electrodes. In a cohort of seven leg amputees, the interface is shown to augment residual muscle afferents by 18% of biologically intact values. Compared with a matched amputee cohort without the afferent augmentation, the maximum neuroprosthetic walking speed is increased by 41%, enabling equivalent peak speeds to persons without leg amputation. Further, this level of afferent augmentation enables biomimetic adaptation to various walking speeds and real-world environments, including slopes, stairs and obstructed pathways. Our results suggest that even a small augmentation of residual muscle afferents restores biomimetic gait under continuous neuromodulation in individuals with leg amputation.

## Abstract (web summary)

Enabled by augmented muscle afferents, a bionic leg under continuous neural control restores biomimetic adaptations to various walking speeds, terrains and perturbations.

## Main

Science fiction has portrayed neurally controlled bionic legs with the same versatility and responsivity as intact biological limbs. Unfortunately, the current state of technology is far less exceptional^1,2^. Current bionic legs rely on predefined robotic control architectures to generate biomimetic locomotion^3–10^. Today’s bionic legs commonly use finite state machine and pattern recognition approaches that model cyclic legged motions into discrete states based upon phase of gait and terrain type. By detecting the current state using robotic sensors, the controller replays predefined intrinsic gait algorithms without continuous neuromodulation from the user.

Neuroprosthetic legs fully driven by the human nervous system, free from reliance on an intrinsic gait controller, may unlock bionic capabilities approaching that of intact limbs. Achieving such functionality would require high-bandwidth neuromodulation to meet gait demands, including adaptative foot positioning, shock absorption and propulsion across diverse terrains^11–14^. Unfortunately, residual motor control in individuals with standard-of-care limb amputation is complicated by inconsistency^15^ and unintended coactivation^16^. Even on level ground, individuals with a standard leg amputation encounter difficulties performing biomimetic gait under continuous neural control^17^. Consequently, contemporary bionic legs use neural inputs only as auxiliary control signals within conventional intrinsic gait controllers^1,2^, typically limited to a specific gait phase and unidirectional movement. Such approaches afford users only partial neural control over gait detection^18^, pattern recognition^19–21^, intrinsic reflex controller gains^22^ or joint movements^9,17^.

The limitations of current systems are not surprising given the complexity of legged neuromechanics. Human gait involves the coordinated interplay between afferent and efferent signals directed to and from the volitional supraspinal and reflexive spinal neural circuitry^12,23,24^. To compound the difficulty, substantial amounts of distal tissue are discarded during the standard-of-care amputation procedure^25^, leading to the loss of essential locomotor peripheral afferents^26,27^.

To compensate for the absence of peripheral afferents, electrical nerve stimulation technology has been proposed and mainly investigated in upper-extremity neuroprostheses^28^. Recent studies^29,30^ demonstrated the effectiveness of direct nerve stimulation to elicit biomimetic afferent responses, resulting in an enhanced upper-extremity neuroprosthetic function. However, locomotion heavily relies on reflexive spinal neural circuitry^12,23,24^, contrasting with upper-extremity motion, and may demand a higher degree of biomimetic afferents because of its limited plasticity compared with supraspinal neural circuits^31^. Moreover, the magnitude of afferents required to facilitate high-bandwidth motor control for the neuromodulation of bionic gait remains elusive. In previous clinical investigations^32–36^, passive and intrinsically controlled bionic legs engineered to provide afferent feedback using electrical nerve stimulation have been shown to improve gait function. However, such systems have yet to demonstrate biomimetic gait when fully driven by the human nervous system.

**Fig. 1:**
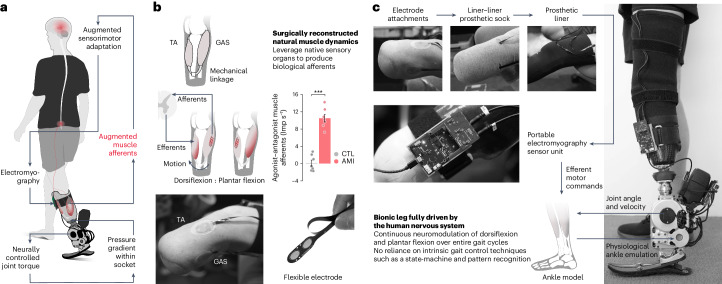
Neuroprosthesis. **a**, Schematic diagram showing the neuroprosthetic interface and a bionic leg fully driven by the human nervous system. Enhancing residual muscle afferents boosted the primary feedback modality for motor gait control and adaptation. Through continuous neural control of the bionic leg, individuals could effectively fine-tune their residual motor control to achieve a biomimetic bionic gait. Mechanical information was conveyed to the nervous system through pressure gradients within the prosthetic socket during ground contact, which mechanically stimulated residual tissues. Such an additional afferent signaling apparatus may provide perceptual experiences for sensorimotor adaptation. **b**, The neuroprosthetic interface consists of the AMI and skin-mounted EMG flexible electrodes. The AMI is shown to augment agonist–antagonist afferent signaling compared with the non-AMI, CTL cohort (bars, mean; error bars, s.e.m.; *n* = 7 per cohort, two-sided unpaired *t*-test, ***P = 7.7 × 10^−7^). Note that four of the seven CTL muscle afferents increased (non-biomimetic) when working as agonists, resulting in negative values for agonist–antagonist muscle afferents. **c**, Components of the autonomous neuroprosthesis are shown. The EMG signals from the TA and GAS were used to continuously control the bionic ankle throughout each phase of gait. To create an upper torque bound for the EMG controller, maximal prosthetic joint torque for each measured joint state was defined using literature values for maximal muscle force–length and force–velocity characteristics at each ankle position and velocity^43,44^. In addition, measured joint state was used to estimate the torque contribution from passive biological structures such as ligaments and joint capsules. Here, no intrinsic gait control techniques were used to drive the bionic leg, such as state machine and pattern recognition algorithms. Extended Data Figs. 1 and 2, respectively, show the neuroprosthetic system and residual muscle neuromechanics in more detail. Source data

Enabled by native peripheral afferents, individuals with biologically intact legs are capable of compensating for locomotory disturbances in seconds^37,38^. Such adaptiveness suggests that augmenting residual afferent signaling may allow individuals with leg amputation to fine-tune their neural circuitry toward a biomimetic neuroprosthetic gait. Muscle afferent signals are known to be one of the primary feedback modalities for functional locomotion^26^. In this study, we therefore hypothesize that augmenting muscle afferents within the amputated residuum will enable an enhanced biomimetic gait adaptation in a neuroprosthetic leg comprising a continuous gait neuromodulation (Fig. 1a).

In this work, we sought to augment residual muscle afferents through a modified amputation, the agonist–antagonist myoneural interface (AMI)^39–41^. The AMI procedure surgically links residual agonist and antagonist muscles to recreate agonist–antagonist muscle dynamics within the amputated residuum. By restoring natural muscle dynamics, the AMI aims to leverage native sensory organs^26,27^ within the residual muscles and tendons to generate biological afferents corresponding to free-space movements of the amputated joints. Our previous reports^40,41^ demonstrated that the below-knee AMI amputation improves volitional free-space control in a benchtop setting. Preliminary results^40^ suggested improved neural control of swing-phase kinematics compared with those without the neuroprosthetic interface. However, in this preliminary investigation the entire stance phase of gait remained under intrinsic control without external neural inputs. Consequently, the bionic gait was not fully driven by the human nervous system. Further, this previous literature does not yet elucidate the relationship between the level of muscle afferent augmentation and the degree of restoration in biomimetic gait neuromodulation.

Here, we evaluate the impact of increasing residual muscle afferents on biomimetic gait neuromodulation in humans with below-knee amputation. We hypothesize that even a small increase in residual muscle afferents will facilitate neural control of biomimetic gait mechanics, adaptability and perturbation response, all without relying on an intrinsic gait controller.

## Results

### Study design and participants

This study was part of an ongoing clinical trial (NCT03913273) assessing the effectiveness of enhancing neuroprosthetic control with the AMI procedure in individuals with transtibial amputation. The AMI amputation involves surgically connecting residual agonist–antagonist muscle pairs to mimic intact biological muscle dynamics after amputation. By emulating the intact muscle dynamics, the AMI aims to utilize the native sensory organs present in the residual muscles and tendons to generate biomimetic afferents that correspond to free-space joint movements (Fig. 1b). For the below-knee AMI amputation, the lateral gastrocnemius (GAS) is mechanically linked to the tibialis anterior (TA) for prosthetic ankle control, and the peroneus longus is linked to the tibialis posterior for prosthetic subtalar control. The inclusion criteria for the study consisted of transtibial amputee subjects aged 18–65 years who demonstrated proficiency using standard passive prostheses and were capable of ambulating with variable cadence (K level 3 and 4 (ref. ^42^)). All participants were required to have fully healed amputation sites, and be free of any pain response that could impact their gait. Exclusion criteria included the following underlying health conditions: cardiopulmonary instability manifested as coronary artery disease, chronic obstructive pulmonary disease and extensive microvascular compromise, as well as pregnancy and/or active smoking. We enrolled seven subjects who had received unilateral below-knee AMI amputation procedures under Partner’s Institutional Review Board (IRB) protocol (p2014001379). An additional seven matched control (CTL) subjects were recruited who had received non-AMI amputations. Before participating in the study, all participants provided written informed consent (IRB protocol 1812634918). The matching criteria between AMI and CTL subjects were subject age, time since amputation, height and weight (Extended Data Table 1). Matching was carried out to assess causal effects by minimizing discrepancies in the group variables. One of the AMI subjects, AMI 2, had previously participated in our pilot study^40^, and had experience with a neuroprosthetic system designed to evaluate the neural control of swing-phase kinematics. However, before participating in this study, all subjects including AMI 2 did not have experience with a bionic system requiring continuous neural control across the entire gait cycle.

In this work, we hypothesize that elevated muscle afferent signaling, facilitated by a neuroprosthetic interface, will promote a highly biomimetic gait under continuous neural control (Fig. 1a). The study was designed to quantify the extent of restored bionic gait functionality fully driven by the human nervous system across varying degrees of augmented muscle afferents. Specifically, we first conducted a residual TA and GAS neuromechanical study to assess subject-specific residual muscle afferents. Subsequently, we performed an array of bionic ambulatory tests, including level-ground walking at multiple speeds, adaptations to slopes and stairs, and obstacle crossing for both AMI and CTL subjects with varying degrees of residual muscle afferents. All patients went through two practice sessions with approximately 6 h of gait experiences in total. Before the data collection, all subjects reported their confidence in performing steady gait. None of the subjects experienced any adverse events such as falling during their participation in the study.

### Neuroprosthesis

**Extended Data Fig. 1:**
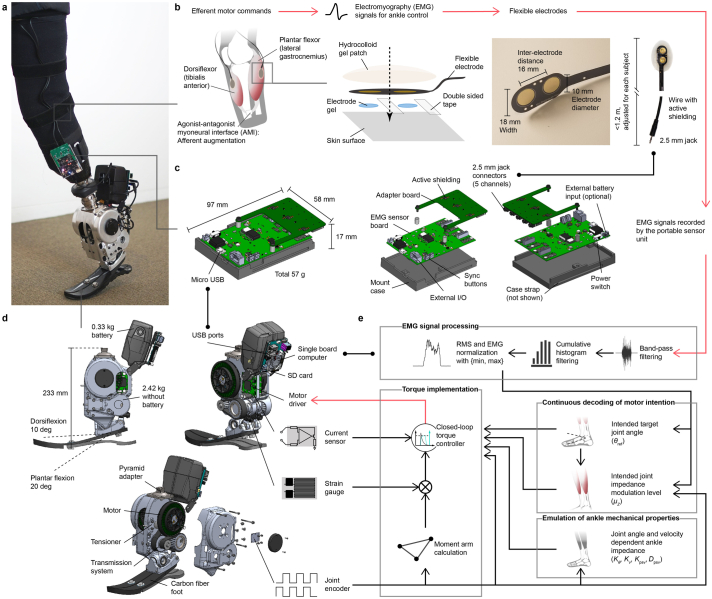
Autonomous bionic technology and control design. **a**, A photograph of the autonomous neuroprosthetic system. **b**, The neuroprosthetic interface for bionic ankle control consists of the Agonist-antagonist Myoneural Interface (AMI) comprising flexible electrodes as the sensing modality for the AMI implementation. **c**, Portable EMG sensor unit. **d**, Autonomous powered prosthetic ankle. **e**, Control design enabling direct neuromodulation of bionic ankle movements. The controller processed the dorsiflexor and plantar flexor electromyography (EMG) signals to compute the EMG envelope. Using the EMG envelope, a decoding pipeline continuously decoded motor intention into target equilibrium joint angle (${\theta }_{{\rm{ref}}}$) and joint impedance modulation level (${\mu }_{Z}$). The ankle actuation direction was obtained through the current joint angle ($\theta$) and ${\theta }_{{\rm{ref}}}$, which was used to account for differences in dorsiflexion (DF)-plantar flexion (PF) motor coordination during the ${\mu }_{Z}$ normalization process. The joint torque command was determined based on an impedance control scheme^78,79^ which modeled joint torque using joint impedance (${K}_{\theta }$, ${K}_{v}$, ${K}_{{\rm{psv}}}$, ${D}_{{\rm{psv}}}$), a neurally-controlled impedance scaling factor ${\mu }_{Z}$, and a neurally controlled equilibrium ${\theta }_{{\rm{ref}}}$. We designed the joint impedance to emulate the biological ankle angle- and velocity-dependent upper torque bound for any measured values of $\theta$ and $\dot{\theta }$. Note that the controller did not involve any intrinsic gait control features like state machines or pattern recognition algorithms for enabling biomimetic gait.

To evaluate our hypothesis, we integrated an autonomous bionic limb consisting of a powered prosthetic ankle, an electromyography (EMG) sensor unit and flexible surface electrodes (Fig. 1 and Extended Data Fig. 1). We designed a neuroprosthetic controller for continuous ankle torque neuromodulation (dorsiflexion (DF) and plantar flexion (PF)) throughout the entire gait cycle, using EMG signals measured from the TA and GAS muscles (Fig. 1c and Extended Data Fig. 1). A single, uniform physiological ankle model was used to compute a target ankle torque value for a given set of EMG signals and prosthetic ankle kinematics. Importantly, the controller used measured prosthetic ankle joint state, or angular position and velocity, to estimate an upper bound for the EMG joint torque controller using empirical measurements from the literature of maximal muscle force–length and force–velocity characteristics at each biological ankle state^43,44^. In addition, measured prosthetic joint state was also used to estimate the torque contribution from passive biological structures such as ligaments and joint capsules. Moreover, it is important to note that the controller did not involve predefined robotic algorithms that rely on knowledge of gait phase, walking speed and underlying terrain type through the use of, for example, pattern recognition algorithms or state machine approaches. Subject-specific, neural-decoding parameters were determined based on EMG profiles during full ankle DF and PF movements of the phantom ankle joint while each subject stood on their biologically intact limb and wore the neuroprosthetic system. Prosthetic system calibration was conducted once at the beginning of the entire session.

### Augmented muscle afferents in residual limbs

**Extended Data Fig. 2:**
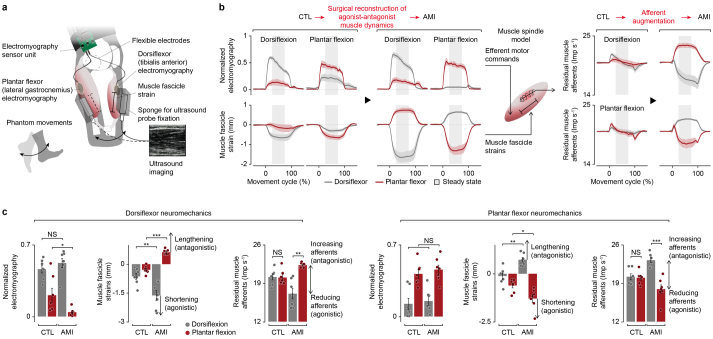
Augmented residual muscle afferents by restored natural muscle dynamics. **a**, Assessment of residual muscle neuromechanics. **b**, Profiles of the recorded EMG, muscle fascicle strains, and estimated muscle afferents for each cohort are reported (*n* = 7 per cohort; bolded lines, mean; shaded regions, SEM). Imp s^-1^, impulses per second. **c**, EMG, muscle fascicle strain, and estimated muscle afferents during the steady state of the phantom ankle movements (bars, mean; error bars, SEM). Larger agonistic shortening and antagonistic lengthening were found in the AMI compared to those of the CTL cohort. The AMI dorsiflexor and plantar flexor produce larger muscle afferents when each muscle works as an antagonist (lengthening) compared to when it works as an agonist (shortening). In contrast, no significant differences are found between dorsiflexor and plantar flexor afferent signaling when each muscle works as an agonist and antagonist in the CTL cohort. For these analyses, two-sided Mann–Whitney U test was used for the GAS EMG comparison during DF, two-sided paired and unpaired *t*-tests for other within and between group comparisons, respectively (*n* = 7 per cohort, *P < 0.035, **P < 0.0068, ***P < 4.9$\times$10^-4^). NS, not significant. Source data

To evaluate muscle afferents in the AMI and CTL cohorts, we examined TA and GAS neuromechanical behaviors during the maximum phantom ankle DF and PF (Extended Data Fig. 2). We estimated muscle afferents using a computational model^45,46^ based on muscle fascicle strains and EMG signals recorded using ultrasound imaging and surface electrodes, respectively. To evaluate an overall muscle afferent signaling for ankle control, we computed agonist–antagonist muscle afferents as the difference between antagonistic (lengthening) and agonistic (shortening) afferents for the TA and GAS muscle pair. We found that the AMI augments agonist–antagonist muscle afferents (AMI: 10.5 ± 0.88 impulses per second (Imp s^−1^), CTL: 0.09 ± 0.69 Imp s^−1^; *n* = 7 per cohort, unpaired *t*-test, *P* = 7.7 × 10^−7^) (Fig. 1b), corresponding to 18% of biologically intact values (approximately 60 Imp s^−1^)^27,45,46^.

### Natural walking with speed adaptation

**Fig. 2:**
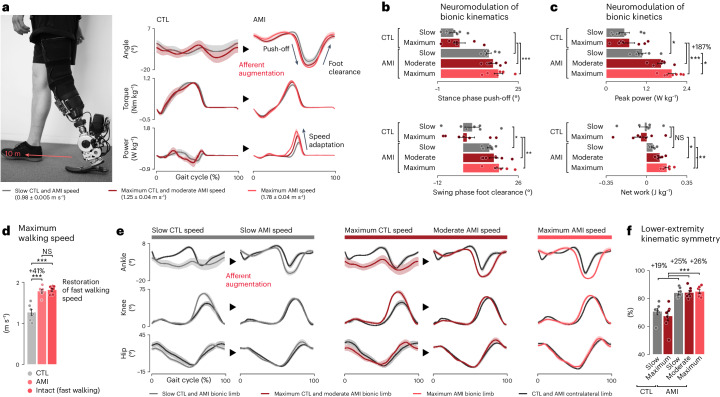
Neuroprosthesis restores biomimetic walking. **a**, Bionic ankle mechanics of level-ground walking in a 10 m open hallway are shown at three walking speeds (*n* = 7 per cohort; bold lines, mean; shaded regions, s.e.m.). Study population walking speeds for each targeted speed are also reported (mean ± s.e.m.). **b**,**c**, Plots report neuromodulated bionic kinematics (**b**) and kinetics (**c**) for each walking speed (bars, mean; error bars, s.e.m.). **d**, The maximum walking speed for each cohort is shown, along with comparisons with the fast walking speeds of biologically intact individuals with similar body weights reported in the literature^50^ (bars, mean; error bars, s.e.m.). **e**, Lower-extremity joint trajectories at three targeted walking speeds are illustrated (bold lines, mean; shaded regions, s.e.m.). **f**, Plot reports LEK symmetry for each targeted speed (bars, mean; error bars, s.e.m.). For these analyses, two-sided paired and unpaired *t*-tests were used for within- and between-group comparisons, respectively, with Holm–Šidák correction for multiple comparisons (*n* = 7 per cohort, *P < 0.049, **P < 0.0096, ***P < 6.2 × 10^−4^). NS, not significant. Extended Data Figs. 3 and 4 report the biomimetic speed adaptation and LEK analyses in more detail, respectively. Source data

We initially tested level-ground walking (Fig. 2a) at a slow speed for all 14 subjects (0.98 ± 0.005 m s^−1^). In addition, we asked each subject to demonstrate steady and stable walking at their self-selected maximum speed. Because AMI subjects generally walked faster than CTL subjects at their self-selected maximum speeds (1.78 ± 0.04 and 1.26 ± 0.07 m s^−1^, respectively), the AMI subjects performed an additional moderate speed walking test to match the maximum speed of the CTL subjects (maximum CTL and moderate AMI population speed: 1.25 ± 0.04 m s^−1^).

**Extended Data Fig. 3:**
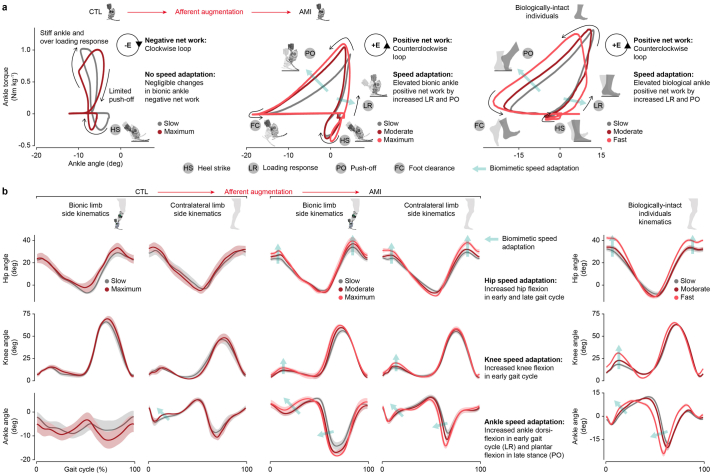
Neuroprosthesis restores biomimetic speed adaptation. **a**, CTL and AMI cohort averaged torque-angle trajectories at various walking speeds are plotted (*n* = 7 per cohort). For comparison, biologically-intact ankle torque-angle trajectories are shown on the right^11^. The AMI cohort torque-angle loop demonstrated a counterclockwise movement, producing positive net work throughout the gait cycle. Moreover, the AMI cohort increased bionic ankle net work by elevating LR and PO as walking speed increased, emulating the speed adaptation mechanics observed in biologically-intact individuals^11^. In contrast, no biomimetic speed adaptation mechanics were found in the CTL cohort. **b**, CTL and AMI cohort lower-extremity kinematics at various walking speeds are plotted (*n* = 7 per cohort; bolded lines, mean; shaded regions, SEM). For comparison, biologically-intact lower-extremity kinematics are shown on the right^11^. The AMI cohort lower-extremity kinematics emulated the speed adaptation found in biologically-intact individuals. In the CTL cohort, speed adaptation was observed only in the contralateral ankle joint kinematics.

We found a higher degree of biomimetic gait in AMI than CTL subjects across multispeed, level-ground walking trials (Fig. 2a and Extended Data Fig. 3). AMI subjects neuromodulated distinct push-off and foot clearance ankle angles to mimic natural joint kinematics (*n* = 7 per cohort, unpaired *t*-tests, *P* < 0.037) (Fig. 2b). In biologically intact limbs, a spinal reflex based on muscle–tendon afferents increases ankle peak power and net work as walking speed increases^47,48^. We found increases in bionic ankle peak power and net work as AMI subjects increased their walking speed (*n* = 7, paired *t*-tests with Holm–Šidák correction, *P* < 0.049 and 0.0042) (Fig. 2c and Extended Data Fig. 3). By contrast, we found no significant change in CTL subject peak power or net work with increasing walking speed (*n* = 7, paired *t*-tests, *P* = 0.073 and 0.11).

AMI subjects demonstrated a 41% higher maximum walking speed compared with CTL subjects (1.78 ± 0.04 and 1.26 ± 0.07 m s^−1^; *n* = 7 per cohort, unpaired *t*-test, *P* = 5.4 × 10^−5^) (Fig. 2d), accompanied by elevated gait energetics, as indicated by a 187% increase in peak power (1.95 ± 0.11 and 0.68 ± 0.17 W kg^−1^; *n* = 7 per cohort, unpaired *t*-test, *P* = 3.1 × 10^−5^) and a 0.223 J kg^−1^ increase in net work (0.174 ± 0.015 and −0.049 ± 0.054 J kg^−1^; *n* = 7 per cohort, unpaired *t*-test, *P* = 0.0018) (Fig. 2c). The elevated gait mechanical energetics restored 65% of prosthetic peak power and net work compared with biologically intact values (3.0 W kg^−1^ and 0.267 J kg^−1^)^49,50^. This level of gait energetic neuromodulation enabled AMI subjects to achieve a walking speed (1.78 ± 0.04 m s^−1^) comparable with that reported for fast walking in individuals with biologically intact legs (1.81 ± 0.03 m s^−1^; *n* = 10 intact and *n* = 7 AMI, Mann–Whitney *U*-test, *P* = 0.52)^50^ having body weights similar to our AMI cohort (69.1 ± 6.0 kg; *n* = 10 intact and *n* = 7 AMI, unpaired *t*-test, *P* = 0.24)^50^ (Fig. 2d).

**Extended Data Fig. 4:**
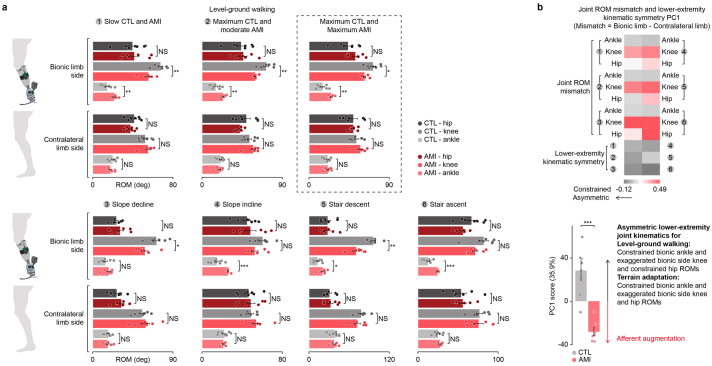
Neuroprosthesis restores symmetric lower-extremity kinematics during gait. **a**, Range of motion (ROM) analysis of lower-extremity joint kinematics for all gait conditions tested. For level-ground walking, both speed-matched and each cohort’s maximum speed walking comparisons are reported. Two-sided Mann–Whitney U test was used for the contralateral knee ROM comparison of maximum CTL and moderate AMI level-ground walking and ankle ROM comparisons of slope adaptation, two-sided unpaired *t*-tests for other comparisons (bars, mean; error bars, SEM; *n* = 7 per cohort, *P < 0.034, **P < 0.0084, ***P < 5.8$\times$10^-4^). NS, not significant. **b**, Principal component analysis (PCA) applied on ROM and symmetry data of lower-extremity kinematics. The maximum speed AMI walking data were excluded from the analysis to focus on data obtained from the matched conditions. The analysis results show that, without the muscle afferent augmentation, CTL subjects increased bionic limb side knee motions to compensate for the constrained bionic ankle, which prevented them from performing symmetric gait. CTL subjects also increased bionic-limb side-hip motions during terrain adaptation to compensate for the constrained bionic ankle. Two-sided unpaired *t*-test was used for the PC1 comparison (bars, mean; error bars, SEM; *n* = 7 per cohort, ***P = 7.0$\times$10^-5^). Source data

The AMI cohort’s neuromodulated, biomimetic, bionic walking mechanics reinstated natural kinematics in the rest of the lower-extremity joints (Fig. 2e and Extended Data Figs. 3 and 4). This resulted in the AMI cohort outperforming CTL subjects in lower-extremity kinematic (LEK) symmetry^51,52^ by 19% to 26% at varying levels of walking speed (*n* = 7 per cohort, unpaired *t-*tests, *P* < 6.2 × 10^−4^) (Fig. 2f). Furthermore, the AMI cohort’s LEK symmetry at the maximum walking speed (84.9 ± 1.3%) was restored to 93% of the LEK symmetry reported for individuals with biologically intact legs (91.4%)^52^.

### Biomimetic terrain adaptation

**Fig. 3:**
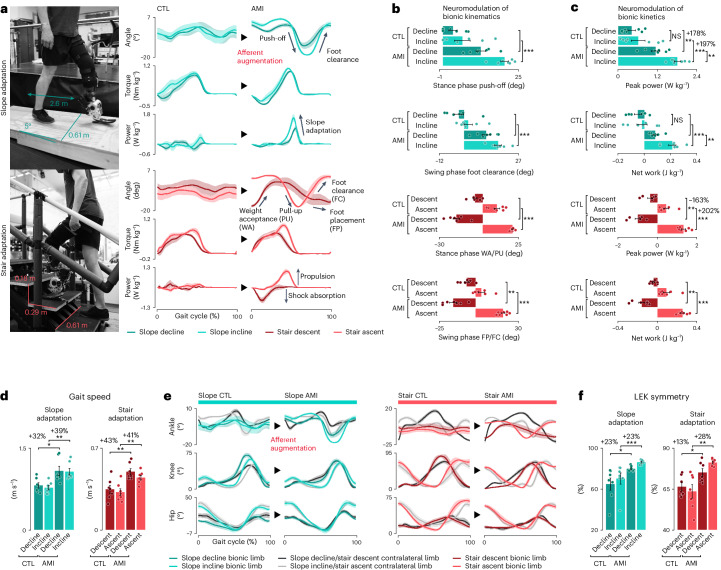
Neuroprosthesis restores biomimetic terrain adaptation. **a**, Bionic ankle terrain adaptation on a 5° slope and staircase is shown (*n* = 7 per cohort; bold lines, mean; shaded regions, s.e.m.). **b**,**c**, Plots report neuromodulated bionic kinematics (**b**) and kinetics (**c**) for each type of terrain adaptation (bars, mean; error bars, s.e.m.). Negative peak power values are reported for stair descent, whereas positive peak power values are reported for all other conditions. Net work during weight acceptance and pull-up is, respectively, reported for stair descent and ascent. **d**, Plots report gait speeds for each type of terrain adaptation (bars, mean; error bars, s.e.m.). **e**, Lower-extremity joint trajectories for each terrain adaptation are illustrated (bold lines, mean; shaded regions, s.e.m.). **f**, Plots report LEK symmetry for each terrain adaptation (bars, mean; error bars, s.e.m.). For these analyses, two-sided Mann–Whitney *U*-tests were used for the gait speed and LEK symmetry comparisons of slope incline, and two-sided unpaired *t*-tests were used for other comparisons (*n* = 7 per cohort, *P < 0.027, **P < 0.0098, ***P < 9.5 × 10^−4^). NS, not significant. Extended Data Figs. 4 and 5 show the LEK analyses and biomimetic terrain adaptation in more detail, respectively. Source data

Individuals with biologically intact limbs can execute versatile gait on various terrains^11–14^. Without changing neuroprosthetic control parameters, we asked all 14 subjects to navigate different terrains, including a 5° slope and stairs (Fig. 3a).

**Extended Data Fig. 5:**
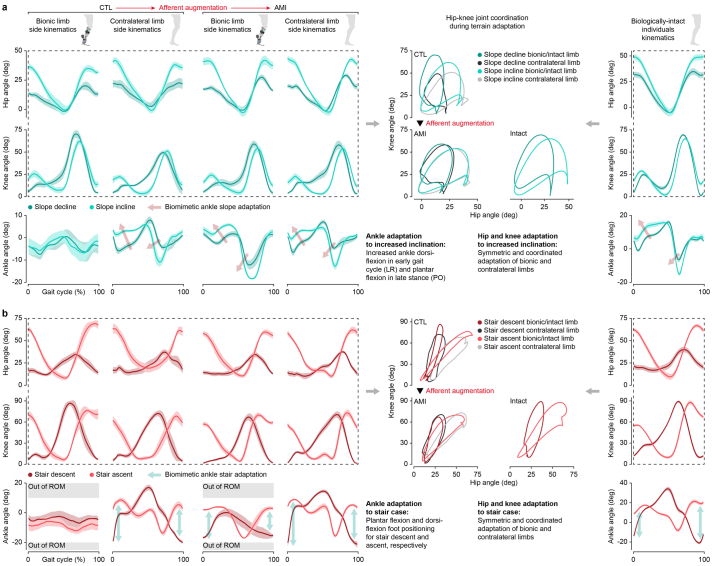
Neuroprosthesis restores biomimetic lower-extremity kinematics during terrain adaptation. **a** and **b**, CTL and AMI cohort lower-extremity kinematics during slope adaptation (**a**) and stair adaptation (**b**) are plotted (*n* = 7 per cohort; bolded lines, mean; shaded regions, SEM). ROM, range of motion. For comparison, biologically-intact lower-extremity kinematics are shown on the right^82^. Averaged hip-knee joint coordination is computed to demonstrate each cohort’s terrain adaptability in detail. The AMI subjects demonstrated highly symmetric and coordinated hip and knee terrain adaptation between limbs, replicating terrain adaptation found in biologically-intact individuals^82^. Additionally, both the bionic and contralateral ankles demonstrated biomimetic foot positioning for effective terrain adaptation^13,14^. In contrast, the CTL subjects showed asymmetric and less coordinated hip and knee terrain adaptation, with only the contralateral ankle demonstrating terrain adaptability. Extended Data Figs. 6 and 7 show the bionic ankle mechanics for biomimetic slope and stair adaptation in more detail, respectively.

For the AMI cohort, the bionic ankle mechanics revealed biomimetic slope and stair adaptive features^12–14^ (Fig. 3a and Extended Data Fig. 5). Notably, AMI subjects maintained the mechanics of level-ground walking during slope adaptation (Fig. 3b) while increasing both peak power and net work for the incline compared with the decline (*n* = 7, paired *t*-tests, *P* = 0.0037 and 0.0045) (Fig. 3c). AMI subjects also modulated shock absorption during initial weight acceptance during stair descent and propulsive torque during the pull-up phase of stair ascent (Fig. 3b,c). This level of versatility was not achieved in previously reported bionic prostheses even with conventional intrinsic control frameworks^1,2^. By contrast, CTL subjects failed to perform biomimetic adaptations on either a slope (*n* = 7, paired *t*-tests, *P* = 0.37 and 0.34) or stairs (Fig. 3b,c). In addition, CTL subjects showed restricted bionic ankle range of motion (ROM) on both slope (*n* = 7 per cohort, Mann–Whitney *U*-test, incline: *P* = 5.8 × 10^−4^) and stair adaptations (*n* = 7 per cohort, unpaired *t*-tests, descent: *P* = 0.034, ascent: *P* = 5.7 × 10^−5^), further demonstrating limited locomotor adaptation capability (Extended Data Fig. 4).

AMI subjects neuromodulated higher positive or negative bionic peak power for adaptative propulsion and shock absorption for each terrain, ranging from 163% to 202% (*n* = 7 per cohort, unpaired *t-*tests, *P* < 0.0052) compared with CTL subjects (Fig. 3c). This broader range of power modulation resulted in biomimetic adaptive gait energetics, as evidenced by the AMI cohort’s increase in net work for propulsion, ranging from 0.133 to 0.232 J kg^−1^, and a 0.12 J kg^−1^ decrease in net work for shock absorption compared with CTL subjects (*n* = 7 per cohort, unpaired *t*-tests, *P* < 0.0041). The ability to neuromodulate biomimetic adaptive gait mechanics enabled AMI subjects to complete terrain adaptation tasks faster, ranging from 32% to 43% (*n* = 7 per cohort, slope incline: Mann–Whitney *U*-test, others: unpaired *t*-tests, *P* < 0.018) compared with CTL subjects (Fig. 3d).

The terrain-adaptive bionic mechanics in the AMI cohort improved kinematics in the rest of the lower-extremity joints (Fig. 3e and Extended Data Figs. 4 and 5). As a result, AMI subjects outperformed CTL subjects in LEK symmetry (*n* = 7 per cohort, incline: Mann–Whitney *U*-test, others: unpaired t-tests, *P* < 0.027), with improvements ranging from 13% to 28% (Fig. 3f).

### Biomimetic perturbation response

**Fig. 4:**
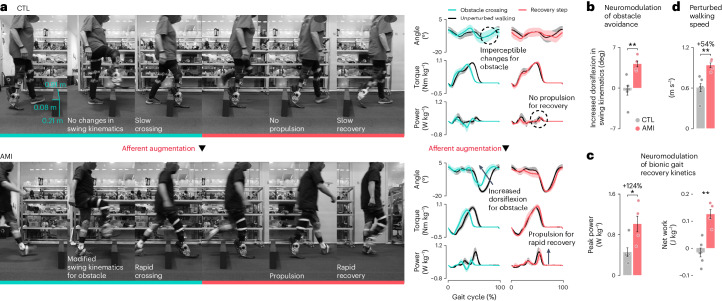
Neuroprosthesis enables a biomimetic perturbation response. **a**, Chronophotography and bionic ankle mechanics of obstacle crossing are shown, including the subsequent recovery step (*n* = 6 CTL and *n* = 4 AMI; bold lines, mean; shaded regions, s.e.m.). Note the increased dorsiflexion angle in swing kinematics in the AMI cohort during obstacle crossing compared with unperturbed walking and propulsive kinetics during gait recovery. **b**, Modified bionic swing kinematics for obstacle crossing are plotted compared with their unobstructed walking values (bars, mean; error bars, s.e.m.). **c**, Plots report neuromodulated bionic kinetics during the subsequent recovery step (bars, mean; error bars, s.e.m.). **d**, Perturbed walking speeds for CTL and AMI cohorts are shown (bars, mean; error bars, s.e.m.). For these analyses, two-sided unpaired *t-*tests were used (*n* = 6 CTL and *n* = 4 AMI, *P = 0.038, **P < 0.0052). Source data

Using a fully neuromodulated bionic limb in real-world conditions requires that the technology respond consistently and safely to complex environmental perturbations^1^. The ability to step over obstacles while walking is a representative perturbation response that involves a close interaction between a supraspinal anticipatory adjustment and spinal reflexive control^53,54^. To test neurally controlled bionic responsivity to environmental perturbations, we conducted obstacle crossing trials (Fig. 4a) with a subset of participants (*n* = 10).

Individuals with biologically intact limbs modify their swing ankle kinematics for obstacle crossing by increasing dorsiflexion compared with their unperturbed walking^54^. Similarly, all AMI subjects increased dorsiflexion in bionic swing kinematics for obstacle crossing compared with their unperturbed walking (4.14 ± 0.66°) (Fig. 4a,b). By contrast, CTL subjects demonstrated imperceptible changes in bionic swing kinematics during obstacle crossing (−0.45 ± 0.87°; *n* = 6 CTL, *n* = 4 AMI, unpaired *t-*test, *P* = 0.0052) (Fig. 4b). Notably, one of the six CTL subjects exhibited a large decrease (−4.22°) in foot dorsiflexion angle compared with their unperturbed walking. This absent or dysfunctional motor response is reported in people living with cerebral palsy^55^ and Parkinson’s disease^56^ and is attributed to disordered supraspinal and spinal locomotor pathways involving peripheral afferents^56–58^. These populations are also known to experience high rates of injury and morbidity from falling^56^.

During the recovery step, AMI subjects produced propulsive power (1.01 ± 0.09 W kg^−1^) and net work (0.125 ± 0.022 J kg^−1^) (Fig. 4c), corresponding to a 124% and 0.141 J kg^−1^ increase compared with CTL subjects (0.45 ± 0.20 W kg^−1^ and −0.016 ± 0.018 J kg^−1^; *n* = 6 CTL, *n* = 4 AMI, Mann–Whitney *U* and unpaired *t*-tests, *P* = 0.038 and 0.0011) (Fig. 4c). This propulsion enabled the AMI cohort to traverse the perturbation at a walking speed of 0.94 ± 0.05 m s^−1^ which was 54% faster than shown by the CTL cohort (0.61 ± 0.06 m s^−1^; *n* = 6 CTL, *n* = 4 AMI, unpaired *t*-test, *P* = 0.0049) (Fig. 4d).

### Augmented afferents restore biomimetic gait control

**Fig. 5:**
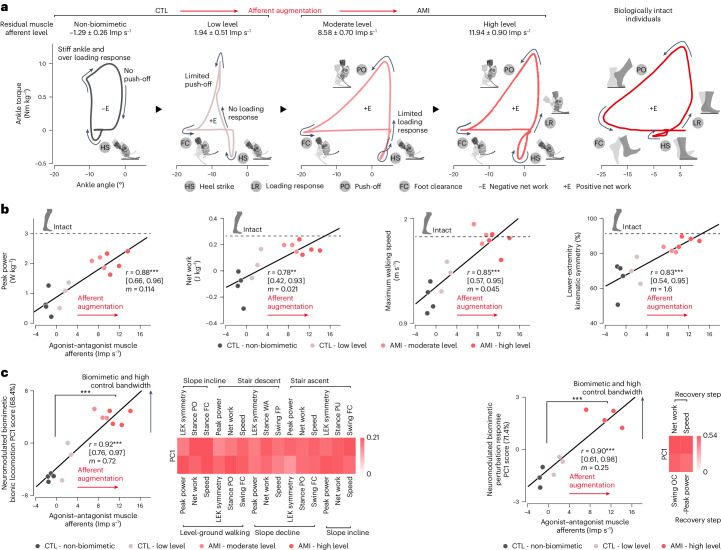
Augmented muscle afferents enable biomimetic gait under neuromodulation. **a**, Plots showing CTL and AMI cohort averaged bionic torque–angle trajectories at maximum walking speeds with varying levels of residual muscle afferents: non-biomimetic (*n* = 4 CTL), low level (*n* = 3 CTL), moderate level (*n* = 3 AMI) and high level (*n* = 4 AMI). Corresponding agonist–antagonist muscle afferents for each group are reported (mean ± s.e.m.). For comparison, a biologically intact ankle torque–angle trajectory is shown on the right^11^. Note how neuromodulated bionic gait evolves toward natural walking as residual muscle afferents increase. **b**, Subjects’ neuromodulated bionic functional metrics at their maximum speeds are plotted against their agonist–antagonist muscle afferents. Pearson correlations (*r*), 95% CI and slopes (*m*) are reported (*n* = 14, **P = 0.0011, ***P < 2.3 × 10^−4^). For comparison, values for each biomechanical metric from a biologically intact population are indicated by dashed lines^49,50,52^. The torque–angle trajectories and correlation plots for other gait conditions are shown in Extended Data Figs. 6–8. **c**, PCA applied to bionic metrics for all gait conditions tested is used to evaluate gross bionic functionality. To have consistent positive signs for biomimetic features, the signs of negative peak power and net work for stair descent are reversed. The PC1 scores are plotted against subjects’ agonist–antagonist muscle afferents. Pearson correlations (*r*), 95% CI and slopes (*m*) are reported (*n* = 14 for unperturbed gait conditions, *n* = 10 for perturbed walking). Two-sided unpaired *t*-tests were used for PC1 comparisons (*n* = 7 per cohort for walking and terrain adaptation, *n* = 6 CTL and *n* = 4 AMI for perturbation response). ***P < 4.4 × 10^−4^. Source data

To understand how a higher degree of biomimetic gait emerges through muscle afferent augmentation, we compared bionic torque–angle trajectories for maximum speed walking at four different muscle afferent levels (Fig. 5a). Individuals’ residual agonist–antagonist muscle afferents were categorized by magnitude as ‘non-biomimetic’ (−1.29 ± 0.26 Imp s^−1^; *n* = 4 CTL), ‘low level’ (1.94 ± 0.51 Imp s^−1^; *n* = 3 CTL), ‘moderate level’ (8.58 ± 0.70 Imp s^−1^; *n* = 3 AMI) and ‘high level’ (11.94 ± 0.90 Imp s^−1^; *n* = 4 AMI).

**Extended Data Fig. 6:**
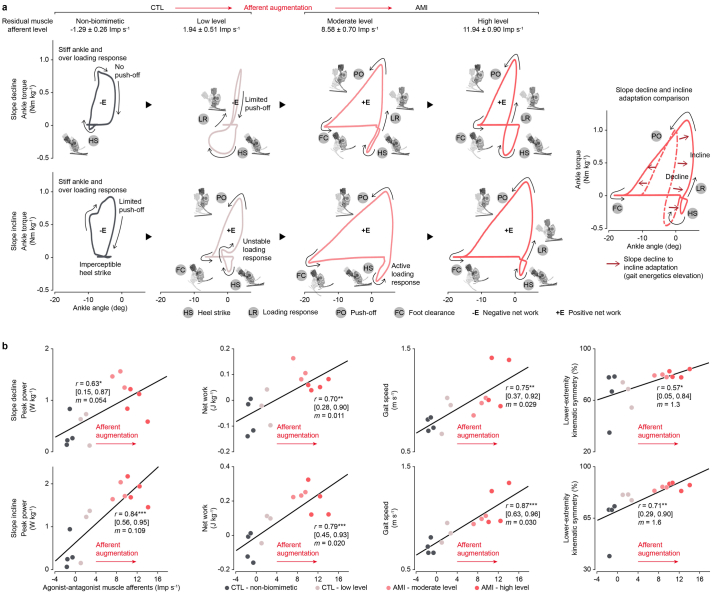
Augmented muscle afferents restore neuromodulation of biomimetic slope adaptation. **a**, CTL and AMI cohort averaged torque-angle trajectories on the 5-degree slope with varying levels of residual muscle afferents are plotted as: non-biomimetic (*n* = 4 CTL), low level (*n* = 3 CTL), moderate level (*n* = 3 AMI), and high level (*n* = 4 AMI). Corresponding agonist-antagonist muscle afferents for each group are reported (mean ± SEM). Note, as residual muscle afferents were augmented, the evolving neuromodulated bionic gait moved towards a natural slope adaptation, regulating gait energetics while maintaining level-ground walking mechanics. **b**, All subjects’ bionic functional metrics in the slope adaptation are plotted against their agonist-antagonist muscle afferents. Pearson correlations ($r$), 95% CI, and slopes (*m*) are reported (*n* = 14, *P < 0.034, **P < 0.0049, ***P < 7.4$\times$10^-4^).

**Extended Data Fig. 7:**
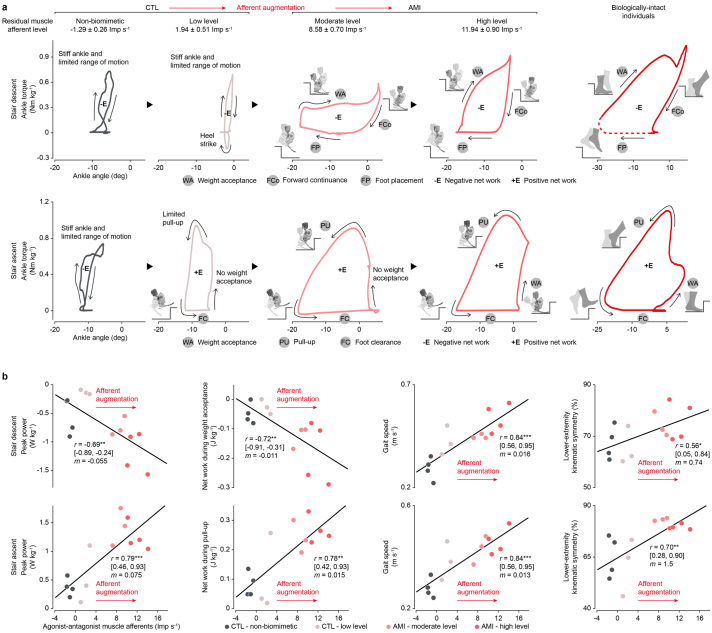
Augmented muscle afferents restore neuromodulation of biomimetic stair adaptation. **a**, CTL and AMI cohort averaged torque-angle trajectories on the staircase with varying levels of residual muscle afferents are plotted as: non-biomimetic (*n* = 4 CTL), low level (*n* = 3 CTL), moderate level (*n* = 3 AMI), and high level (*n* = 4 AMI). Corresponding agonist-antagonist muscle afferents for each group are reported (mean ± SEM). For comparison, a biologically-intact ankle torque-angle trajectory is shown on the right^11^. Due to the lack of data points for the torque-angle trajectory plot, the dotted line for the biologically-intact limb plot extends from the original data^11^. Note, as residual muscle afferents were augmented, the evolving neuromodulated bionic gait moved toward natural stair adaptation. **b**, All subjects’ bionic functional metrics in the stair adaptation are plotted against their agonist-antagonist muscle afferents. Negative peak power values are reported for stair descent, whereas positive peak power values are reported for stair ascent. Net work during weight acceptance and pull-up are reported for stair descent and ascent, respectively. Pearson correlations ($r$), 95% CI, and slopes (*m*) are reported (*n* = 14, *P = 0.035, **P < 0.0069, ***P < 6.8$\times$10^-4^).

For the ‘non-biomimetic’ afferents, the torque–angle loop was not biomimetic, moving in a clockwise direction and dissipating mechanical energy from the gait cycle (Fig. 5a). In distinction, as residual muscle afferents were augmented from ‘non-biomimetic’ to ‘high level’ levels, the torque–angle loops illustrated the emergence of natural walking mechanics, moving in a counterclockwise direction and injecting net positive work into the walking gait cycle (Fig. 5a). The level of afferent augmentation was also strongly correlated (*r*) with subjects’ bionic ankle peak power (*r* = 0.88, *n* = 14, *P* = 3.2 × 10^−5^), net work (*r* = 0.78, *n* = 14, *P* = 0.0011), LEK symmetry (*r* = 0.83, *n* = 14, *P* = 1.3 × 10^−4^), maximum walking speed (*r* = 0.85, *n* = 14, *P* = 2.3 × 10^−4^) and agonist–antagonist muscle afferents (Fig. 5b). Biomimetic slope and stair adaptations also gradually emerged as muscle afferents were augmented (|*r*|= 0.56–0.87, *n* = 14, *P* < 0.035) (Extended Data Figs. 6 and 7 and Extended Data Table 2). These results demonstrate that muscle afferent augmentation enhances overall neuromodulated bionic limb functionality (*r* = 0.92, *n* = 14, *P* = 3.6 × 10^−6^) (Fig. 5c).

**Extended Data Fig. 8:**
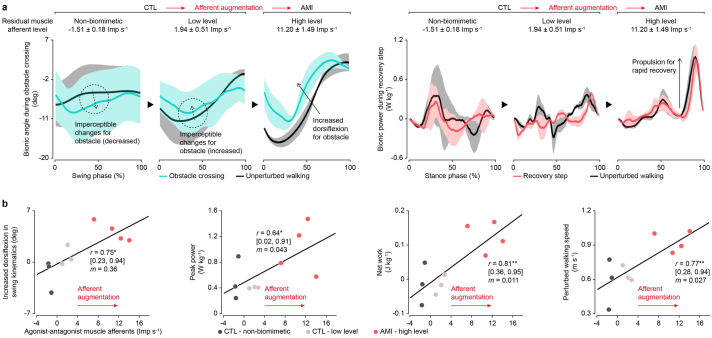
Augmented muscle afferents restore neuromodulation of biomimetic response to perturbation. **a**, With varying levels of residual muscle afferents for CTL and AMI cohorts, the modified swing kinematics during obstacle crossing and stance kinetics during a recovery step are plotted as: non-biomimetic (*n* = 3 CTL), low level (*n* = 3 CTL), and high level (*n* = 4 AMI) (bolded lines, mean; shaded regions, SEM). Resulting from the augmented residual muscle afferents, note the emerging neuromodulated biomimetic swing kinematics for obstacle crossing and stance kinetics during the recovery step. **b**, All subjects’ bionic perturbation response metrics are plotted against their agonist-antagonist muscle afferents. Pearson correlations ($r$), 95% CI, and slopes (*m*) are reported (*n* = 10, *P < 0.046, **P < 0.0089).

Similarly, as the magnitude of residual muscle afferents was elevated, subjects were able to distinctively modify swing kinematics for obstacle crossing, generate propulsive gait kinetics for the subsequent recovery step and consequently walk faster under perturbation (*r* = 0.64–0.81, *n* = 10, *P* < 0.046) (Extended Data Fig. 8 and Extended Data Table 2). This result shows that muscle afferent augmentation enables a biomimetic and rapid bionic response to perturbation (*r* = 0.90, *n* = 10, *P* = 4.4 × 10^−4^) (Fig. 5c).

Considering the magnitude of augmented residual muscle afferents (10.50 ± 0.88 Imp s^−1^, *n* = 7 AMI) compared with intact values (approximately 60 Imp s^−1^)^27,45,46^, our results suggest that even a modest augmentation in residual muscle afferents restores the amputee’s capability to neuromodulate biomimetic and versatile locomotion, and to respond to real-world environmental perturbations.

## Discussion

In this study we show that residual muscle–tendon afferents enable a person with transtibial amputation to directly neuromodulate biomimetic locomotion, enabling neuroprosthetic adaptations to varying walking speeds, terrains and perturbations. Such versatile and biomimetic gait has not been attainable in contemporary bionic legs without the reliance upon predefined intrinsic control frameworks^1,2^. Central to the improved neural controllability demonstrated in this study are muscle–tendon sensory organs^26,27^ that deliver proprioceptive afferents. The surgically reconstructed, agonist–antagonist muscles emulate natural agonistic contraction and antagonistic stretch, thereby generating proprioceptive afferents corresponding to residual muscle movements.

During the ground contact phase of walking, the reconstructed muscle–tendon dynamics of the AMI do not precisely emulate intact biological muscle dynamics. The residual muscles of the AMI contract and stretch freely within the amputated residuum, only pulling against one another and not against the external environment. In distinction, for intact biological limbs, the muscle–tendons span the ankle joint, exerting large forces through an interaction with the external environment. These interactive muscle–tendon dynamics in intact biological limbs are believed to play a critical role in spinal reflexes, in addition to providing feedback for volitional motor control^12^. Therefore, for this study, the demonstrated capacity of augmented afferents to enable biomimetic gait neuromodulation is surprising given that their total magnitude is largely reduced compared with those of intact biological limbs^26,27,45,46^. We thus surmise that humans exhibit extensive sensorimotor adaptability, wherein even a small amplification of residual muscle–tendon afferent signaling can effectively fine-tune both volitional supraspinal and reflexive spinal neural circuitry to enable biomimetic gait neuromodulation. The relationships found in this investigation between the reconstructed residual muscle–tendon morphology and the observed neuromodulated bionic functionality offer design principles for future neuroprosthetic interfaces. By considering limb reconstruction procedures as a neuroprosthetic design optimization problem, our results enable more-effective approaches to enhancing bionic limb function after amputation.

Contemporary bionic legs rely on intrinsic control systems to guide biomimetic gait^1,2^. Such intrinsic controllers estimate gait phases, speeds and environmental conditions, and then model diverse legged motions into a finite number of states. Indeed, accurately estimating such gait information poses technical challenges^10^, and in principle, modeling human motor intent into a finite number of states inevitably results in limiting the native versatility of legged locomotion. The full neural control scheme presented in this study instead relies on the human nervous system to directly neuromodulate the bionic leg to adapt across gait phases, speeds, environments and perturbations. Our efforts focused on augmenting an individual’s residual motor control and sensorimotor adaptation to a bionic limb, while regarding the external bionic limb itself as a mere physical emulator of the lost limb without dominant intrinsic control authority.

The use of passive ankle–foot prostheses by persons with transtibial amputation causes non-biomimetic gait mechanics, resulting in less-stable gait with secondary complications such as increased energy expenditure^59,60^, osteoarthritis^61,62^, osteoporosis^63^ and lower back pain^64^. Compared with passive devices, intrinsically controlled powered prostheses improve gait mechanics and mediate these complications^4,65,66^. Moreover, prosthetic efferent control and sensory feedback are recognized as contributing factors to the enhancement of prosthesis embodiment^67,68^. Therefore, we anticipate similar clinical advantages and improved embodiment stemming from a neuroprosthesis fully driven by the human nervous system with augmented residual muscle afferents. Future studies should focus on demonstrating the clinical benefits of long-term usage of neuroprostheses with afferent augmentation compared with prescribed passive and intrinsically controlled powered prostheses. In addition, future studies that focus on patient experiences, such as potential improvements in confidence, cognitive load and embodiment, could provide a better understanding of the benefits offered by neuroprostheses in the realm of users’ daily lives and psychological well-being.

Despite the external bionic leg used in this study having a comparable mass (2.75 kg) to the missing limb (approximately 2.89 kg; foot and half of the shank: 3.7% of body weight^69^), subjects initially reported the bionic leg to feel heavy during the early stages of the gait trial, presumably because of the large weight difference from their prescribed passive prosthesis. Interestingly, as the gait trial progressed, subjects that exhibited a higher degree of biomimetic gait patterns commented that the neuroprosthesis no longer felt heavy. This experience might be attributed to the biomimetic ankle dynamics compensating for prosthetic mass, and to the enhancement of prosthetic embodiment^68^ promoted by the augmented afferents.

In this work, we mainly focused on the capacity of the muscle–tendon afferents to enable biomimetic gait neuromodulation. However, previous literature^32–36^ on passive and intrinsically controlled prosthetic gait suggests that adding cutaneous feedback could further improve bionic functionality. Further, surgical interventions such as osseointegration^70^ could also provide useful afferents (for example, osseoperception^71^) in addition to a better electromechanical coupling between the residuum and external bionic limb.

In this study, we investigated the ability for individuals with the AMI limb amputation to neuromodulate biomimetic locomotion across a wide range of bionic ambulatory tests. Nevertheless, to fully address bionic versatility and responsivity akin to intact biological limbs, bionic capabilities need to be tested under a broader set of locomotory conditions. Such future testing might include motor tasks that involve higher control bandwidths, such as sprinting, jumping and single-leg balancing. Moreover, this study only explored bionic responsivity to a static environmental perturbation. Future studies might demonstrate bionic responsivity to more dynamic perturbations, such as randomized rough terrains and balance perturbations.

To date, the AMI amputation procedure has been performed on more than 50 patients at various amputation levels, including transtibial, transfemoral, transradial and transhumeral levels. In future studies, we plan to assess the capacity of these surgical design techniques to enhance residual afferents and improve neuroprosthetic control at multiple degrees of freedom and alternate amputation levels. The new findings of this investigation may inform further advancements in surgical reconstruction and neural interfacing technologies to achieve even greater levels of afferent restoration and biomimetic motor function. We also expect that similar technological frameworks can be applied to the neuroprosthetic integration of a broad spectrum of wearable devices ranging from upper-extremity prostheses to exoskeletal limbs. Given the technological challenges associated with complete afferent restoration, our findings suggest that partial reinstatement may be sufficient for the human nervous system to achieve biomimetic neuroprosthetic function under full neuromodulation.

## Methods

### Experimental design

This study was part of our ongoing registered clinical trial (NCT03913273), which seeks to assess the impact of the AMI amputation procedure in persons with transtibial amputation. The AMI amputation involves surgical reconstruction of the residual limb with an intent to emulate agonist–antagonist muscle dynamics and to leverage muscle–tendon sensory organs to preserve afferent proprioceptive signaling (Fig. 1b). The study follows a prospective, nonrandomized design. Neither sex nor gender were considered in this study. We recruited 14 participants (Extended Data Table 1), 7 of whom had received the AMI amputation^40^ (AMI cohort) and 7 of whom had received a non-AMI amputation^25,72^ (CTL cohort). Sex was self-reported. The matching criteria between AMI and CTL subjects were subject age, time since amputation, height and weight. We hypothesized that enhancing muscle–tendon afferents within the amputated residuum would enable biomimetic gait fully driven by the human nervous system. To test our hypothesis, we: (1) developed an autonomous bionic system offering continuous neural control; (2) evaluated gait using a wide range of ambulatory tests including level-ground walking at multiple speeds, adaptations to slopes and stairs, and obstacle crossing; (3) performed a neuromechanical study to assess subject-specific levels of residual limb muscle afferents; and (4) conducted correlation analyses between subject-specific bionic functionality and residual limb muscle afferents. Further, we conducted statistical group comparisons between the AMI and CTL cohorts to demonstrate efficacy of the proposed neuroprosthetic framework. To address the clinical trial aim of determining whether AMIs can improve prosthetic terrain adaptations, we assessed two predefined outcome measures: swing-phase PF control during stair descent and swing-phase DF control during stair ascent.

### Participants

Fourteen participants with unilateral below-knee amputation (age 47.6 ± 3.5 years, time since amputation 3.9 ± 0.5 years, height 1.73 ± 0.02 m, weight 78.1 ± 3.2 kg) participated in this study. Sample size was chosen on the basis of data from our previous study^41^. The participants and research team were not blinded to the testing conditions. All participants were proficient in the use of standard passive prostheses and were capable of ambulation with variable cadence (K level 3 and 4 (ref. ^42^)). All participants had fully healed amputation sites and reported no pain that could affect their gait. Exclusion criteria included the following underlying health conditions: cardiopulmonary instability manifest as coronary artery disease, chronic obstructive pulmonary disease and extensive microvascular compromise, as well as persons who are pregnant and/or active smokers. Before participating in the study, all participants provided written informed consent. The participants were compensated on an hourly basis for the study (US$20 per hour). The study was IRB-approved by our Committee on the Use of Humans as Experimental Subjects (protocol 1812634918) at the Massachusetts Institute of Technology (MIT). The study protocol can be made available upon reasonable request to the corresponding authors. The period of recruitment and data collection was from April 2019 to January 2023. All data were collected at the MIT, Massachusetts.

### Estimation of residual limb muscle afferents

To evaluate subjects’ residual limb muscle afferents, residual dorsiflexor (TA) and plantar flexor (GAS) neuromechanics were investigated during ten cycles of each maximum phantom DF and PF (Extended Data Fig. 2). The surface EMG and the corresponding TA and GAS muscle fascicle strain data were simultaneously recorded by our custom EMG sensor system^73^ and a commercially available ultrasound sensor (LS128, Telemed), respectively. A flexible electrode form factor allowed us to simultaneously record EMG and muscle fascicle strain data even when limited skin surface area was available^74^. The fascicle strain was computed from the ultrasound video recordings using UltraTrack software^75,76^. Raw EMG signals from residual TA and GAS were band-passed with a finite impulse response filter (stop-band: 0–60 Hz, >360 Hz; pass-band: 90–330 Hz, a stop-band attenuation of 85 dB, order: 198). The root-mean-square of rectified EMG (200-ms window size) was then normalized by using its minimum and maximum values to compute the EMG envelope. The muscle fascicle strains and EMG envelopes were normalized by the movement cycle to allow intrasubject and intersubject analyses. Averaged muscle fascicle strain and EMG envelope profiles for ten phantom DF and PF cycles were used for the muscle afferent estimation. We estimated muscle afferents using a computational model (type II muscle spindle model^45,46^). We used average muscle afferent values during steady states of maximum phantom DF and PF (between 25% and 75% of the cycle) to evaluate TA and GAS afferents. We computed the bandwidth of residual limb muscle afferent signaling (agonist–antagonist muscle afferents) as the difference between the antagonistic (lengthening) afferents and the agonistic (shortening) afferents of the muscle pair.

### Bionic system and integration

The bionic limb used in this study (Extended Data Fig. 1) consisted of a powered prosthetic ankle, portable EMG sensor unit and flexible electrodes developed in our group^73,77^. The powered prosthetic ankle generated active joint torque at a maximum of 162 newton meters (Nm) DF–PF torque using two brushless electric motors (U8 Lite KV85, T-motor). The ROM of the powered prosthetic ankle was 10° of DF and 20° of PF. The ankle–foot prosthesis was 233 mm in height and weighed 2.42 kg without a battery module. The battery module weighed 0.33 kg and could power the bionic system for approximately 2 h under continuous usage with a single charge. The flexible bipolar surface electrodes (width: 18 mm, length: 60 cm) were fabricated using flexible printed circuit board technologies. Because of flexibility and film thickness (80–100 μm), the electrodes allowed recording EMG without causing discomfort or pain during the long-duration gait trial. The portable EMG sensor system (weight: 57 g) provided up to five channels for simultaneous EMG recording at 2 kHz with active shielding.

The system integration procedure was as follows (Fig. 1c and Extended Data Fig. 1). In step 1, we placed one flexible bipolar surface electrode on each target muscle (TA and GAS). We used standard double-sided tape, electrode gel (SPECTRA 360 electrode gel, Parker Labs Inc) and hydrocolloid gel patch (blister bandages, Pnrskter) to attach the electrodes to the targeted sites. In step 2, we guided the flexible electrodes and their lead wires through liner–liner prosthetic socks to minimize direct contact between the electrodes and skin surfaces, thereby mitigating potential skin damage due to friction. In step 3, the subject put on the prosthetic liner while keeping the flexible electrodes flat and untangled. In step 4, the subject then donned their customary socket, and we attached the powered ankle–foot prosthesis to the socket using a standard pyramid adapter. In step 5, we plugged the flexible electrodes into the EMG sensor unit, and strapped the unit to the side of the socket. Finally, in step 6, the EMG board was connected to the powered ankle–foot prosthesis using a universal serial bus.

### Bionic control design

The goal of our bionic control design was to enable the user’s nervous system to continuously modulate bionic joint torques across a versatile array of motor tasks. The controller did not involve predefined robotic algorithms that rely on knowledge of gait phase, walking speed and underlying terrain type. In this framework, the bionic limb emulated the lost limb functionality without an intrinsic control authority used as an alternative to the user’s own nervous system control.

The control design was achieved based on an impedance control architecture^78,79^ using EMG from residual TA and GAS, as well as bionic joint angle and velocity information (Extended Data Fig. 1).

Raw EMG signals from residual TA and GAS were band-passed with a finite impulse response filter (stop-band: 0–60 Hz, >360 Hz; pass-band: 90–330 Hz, a stop-band attenuation of 85 dB, order: 198) and cumulative histogram filter^80^ to facilitate robust reading within the liner-socket system. Then, root-mean-square of rectified EMG (200-ms window size) was normalized by using its minimum and maximum values to compute the EMG envelope. We computed muscle activity from the EMG envelope using bilinear muscle activation dynamics^81^.

The controller decoded motor intention from residual TA and GAS muscle activities into two control variables: target equilibrium joint angle (${\theta }_{{\rm{ref}}}$) and joint impedance modulation level (${\mu }_{Z}$). The value of ${\theta }_{{\rm{ref}}}$ was computed by the weighted muscle pair activity difference and filtered by a critically damped mass-damper-stiffness model (second order low-pass filter, cut-off frequency 6 Hz). The weighted coefficients of ${\theta }_{{\rm{ref}}}$ for each subject were determined such that the subjects’ muscle activities during maximal phantom DF and PF activations were mapped to the powered prosthetic ankle’s maximum DF and PF. These maximal DF and PF activations were collected while each subject stood on their biologically intact and bionic limbs. The value of ${\mu }_{Z}$ was computed by normalizing the sum of the weighted muscle pair activity such that it varied between 0 and 1. Because of differences between DF and PF motor coordination, we used two different values for the ${\mu }_{Z}$ normalization during each bionic DF and PF actuation. To mimic the biological ankle’s angle-dependent and velocity-dependent torque characteristics, the bionic torque was determined based on: (1) the difference ($\delta$) between ${\theta }_{{\rm{ref}}}$ and the actual prosthetic ankle angle; (2) ${\mu }_{Z}$; and (3) prosthetic joint angle and velocity. For the maximum DF or PF motor intention, indicated by ${\mu }_{Z}$ = 1 and maximum $\delta$, the torque command was determined by the maximum angle-dependent and velocity-dependent biological ankle torque^43,44^ corresponding to the measured prosthetic joint angle and velocity. For nonmaximum motor intention, the joint torque command scaled the maximum torque value by the factor of ${\mu }_{Z}\delta$. Additional virtual damping (0.02 Nm s degree^−1^) and stiffness (0.45 Nm degree^−1^) were added to ensure bionic joint stability under zero muscle activity. The torque command was achieved by proportional feedback control with damping injection based upon intrinsic sensor readings. See the Supplementary Information for detailed formulation.

### Level-ground walking and terrain adaptation experiments

To ensure participant safety, the subjects performed an initial bionic adaptation on a 3.6-m walkway with handrails. The subjects were advised to use handrails whenever needed to help balance, walk at self-selected speed or pause. The subjects were further advised to neuromodulate the bionic gait in a way that felt natural to them. However, in an effort to investigate sensorimotor integration and controllability of the bionic limb based solely on afferent and efferent neural signaling, we did not give the subjects any detailed guidance on how to achieve the biomimetic gait during any experimental sessions. Once a subject was confident and able to perform steady and stable walking, we proceeded as follows.

Two practice sessions were conducted before starting data collection. Each practice session was performed in the order of level-ground walking, slope adaptation and stair adaptation, thereby gradually increasing the difficulty of gait to ensure subject safety. The goal of the practice sessions was to provide sufficient experience to allow steady and stable gait using the bionic limb. During the level-ground walking trial, each subject was guided to walk along a 10-m open hallway (10-m walk test). The travel time was recorded to evaluate walking speed. We tested level-ground walking for all subjects at a slow walking speed (the targeted speed and travel time were, respectively, 1.0 m s^−1^ and 10 s). In addition, we asked each subject to demonstrate steady and stable walking at their self-selected maximum speed. Because we found the AMI subjects generally walked faster than CTL subjects at their self-selected maximum speeds, we additionally tested the AMI subjects at a moderate walking speed matching the CTL subjects’ maximum walking speed (the targeted speed and travel time were, respectively, 1.25 m s^−1^ and 8 s). The research team cued the subject to please ‘speed up’ or ‘slow down’ if their travel time did not meet the targeted walking speed. Acceptance criteria for each targeted speed trial was a 1-s window from the targeted travel time (±0.5 s).

Four types of terrain adaptation trials were conducted including 5° slope decline/incline and stair descent/ascent. For each terrain adaptation, the subjects were told to initiate their gait at a self-selected speed. The research team advised the subjects to use handrails whenever needed to help them balance, perform gait or pause. The subjects were asked to try to gradually reduce the usage of the handrails and speed up until they identified the maximum speed at which they felt they could safely maintain a steady gait. The gait speed for each terrain adaptation was calculated from the travel distance and time.

One hour was assigned as a gait acclimatization period for each level-ground, slope and stair walking adaptations, resulting in an approximately 6 h of gait experience for all subjects with at least an additional 15 min of rest between conditions. Further, the subjects were allowed to rest upon request to minimize fatigue. All subjects stated that they were confident in performing stable and steady gait before the start of data collection.

### Bionic gait data collection and functional metrics

Bionic ankle kinematics, torque and ipsilateral and contralateral LEK were simultaneously recorded using onboard sensors and goniometers (wireless goniometer, Biometrics Ltd), and sent by telemetry to a laptop at 1 kHz. We calculated bionic ankle power and net work from the recorded bionic joint kinematic and torque data. Biomimetic stair adaptive features are shock absorption during the stair descent weight acceptance and propulsive torque during the stair ascent pull-up phase^13^. Therefore, we used negative peak power values for the stair descent analysis and positive peak power values for analyses of all other gait conditions. Net work during weight acceptance and pull-up were used for stair descent and ascent analyses, respectively. Fifteen gait cycles were collected for each level-ground walking condition. Ten gait cycles were collected for each terrain adaptation trial. The gait data were normalized by gait cycle, and prosthetic ankle torque, power and net work were further normalized by subject mass to allow intrasubject and intersubject analyses. We qualitatively evaluated bionic ankle mechanics through torque–angle trajectory analyses^3,17^ and compared bionic gait performances with biologically intact gait using publicly available data^11,49,50,52,82^.

### Bionic kinematic analysis based on gait events

We assessed bionic ankle behaviors based on gait events using each participant’s average joint kinematic trajectories. We subdivided level-ground walking and slope adaptation gait events into heel strike, loading response, push-off (PO) and foot clearance (FC)^14,83^. We computed heel strike by PF from initial ground contact to the first local maximum PF during stance. We calculated loading response by DF from the first local maximum PF to the maximum DF during stance. PO was evaluated by PF from the maximum DF during stance to the joint angle at the end of stance. FC was evaluated by DF from the joint angle at the end of stance to the joint angle at the end of swing. We subdivided stair descent gait events into weight acceptance (WA) and forward continuance/foot placement (FCo)^13,84^. For stair ascent, we subdivided gait events into WA, pull-up (PU) and FC. We computed WA by DF from initial ground contacts to the maximum DF during stance. We evaluated FCo of stair descent by PF from the maximum DF during stance to joint angle at the end of swing. We evaluated PU and FC during stair ascent in the same way as PO and FC during level-ground walking and slope adaptation. As representative metrics for neuromodulated bionic kinematics, we selected the critical gait phases for propulsion, shock absorption and foot positioning. These included PO and FC for level-ground walking and slope adaptation; WA and FCo for stair descent; and PU and FC for stair ascent.

### Lower-extremity kinematic symmetry

LEK symmetry was evaluated through a mean value of symmetry index^52^ (SI) across ankle, knee and hip joints integrated over a full gait cycle. Because the SI is an index for kinematic asymmetry^52^, we computed kinematic symmetry using the equation 100 − SI (%).

### Range of motion for lower-extremity kinematics

The ROM for ipsilateral and contralateral lower-extremity joints was computed as the difference between the maximum DF and PF, or maximum flexion and extension, in each participant’s average joint kinematic trajectories for each gait testing condition. We applied a principal component analysis (PCA) over ROM mismatch between bionic and contralateral limb joints, and LEK symmetry for level-ground walking and terrain adaptation. The metrics were normalized using their mean and standard deviation before applying PCA. We used the first principal component (PC1) and PC1 score to assess subjects’ overall strategy of using lower-extremity joints to execute target locomotion.

### Perturbed walking experiments

To test the neuromodulated bionic responsivity to an environmental perturbation, we performed an exploratory perturbed walking trial. Because of limited participant availability and scheduling challenges, the additional perturbed walking trial was conducted with a subset of participants (*n* = 10, CTL 1–6 and AMI 1–4; Extended Data Table 1). We assessed bionic ankle behavior during the obstacle step crossing and the recovery step. We used a stiff sponge that was 0.21-m high and wide enough to obstruct the subjects’ path as the obstacle (thickness 0.08 m, width 0.91 m). Participants were advised to walk at their maximum self-selected speed at which they could traverse their obstructed path at a steady pace. Twelve gait cycles were collected for each obstacle crossing, with the bionic ankle leading during the recovery step. Corresponding walking data without obstacle crossing were also collected to provide unperturbed walking baselines for within subject analyses. The neuromodulated bionic response for the obstacle crossing was assessed by computing the average increase in DF of the bionic swing kinematics compared with that of unperturbed walking. We adopted this index as it better represents neurally controlled perturbation responses than the widely used minimum distance between foot and obstacle, which does not necessarily correlate with neuromodulated bionic responses. We used the bionic ankle peak power and net work during the recovery step to evaluate the propulsive mechanics for rapid gait recovery. A video camera (EOS 5D Mark III, Canon) recorded the sagittal plane view of an individual performing perturbed walking to estimate the travel distance from the start of the obstacle crossing swing phase to the end of the recovery step stance phase. The perturbed walking speeds were computed from the travel distance and time.

### The influence of residual muscle afferents on bionic torque–angle behavior

To investigate how augmented muscle afferents contributed to biomimetic gait neuromodulation, we computed averaged torque–angle trajectories^3,17^ at four different levels of residual muscle afferents. Among 14 participants, the 4 subjects who indicated lowest and negative agonist–antagonist muscle afferents (*n* = 4; CTL 1, 3, 6, 7) were grouped as ‘non-biomimetic’. The next three subjects who indicated low agonist–antagonist muscle afferents (*n* = 3; CTL 2, 4, 5) were grouped as ‘low level’. Similarly, the ‘moderate level’ and ‘high level’ consisted of the next three (*n* = 3; AMI 4, 5, 6) and four subjects (*n* = 4; AMI 1, 2, 3, 7), respectively, by the order of agonist–antagonist muscle afferent values. For the level-ground walking analysis, the trajectories at the maximum walking speed were used to evaluate each individual’s maximum walking capability. We analyzed the torque–angle trajectories to assess overall gait cycle dynamics and mechanical energetics.

### Correlation analysis between bionic functionality and muscle afferents

To investigate the relationship between subject-specific bionic gait controllability and residual limb muscle afferents, we plotted all subjects’ peak power, net work, gait speeds and LEK symmetry against their agonist–antagonist muscle afferents for each steady gait testing condition. For the level-ground walking analysis, the values of subjects’ maximum walking speeds were used to evaluate relationships between their maximum walking capability and varying levels of agonist–antagonist muscle afferents. The four residual muscle afferent groups are determined as described in the bionic torque–angle analyses section described earlier. Similarly, the three perturbed walking analysis groups were defined by the magnitude of the agonist–antagonist muscle afferent values, consisting of ‘non-biomimetic’ (*n* = 3; CTL 1, 3, 6), ‘low level’ (*n* = 3; CTL 2, 4, 5) and ‘high level’ (*n* = 4; AMI 1–4). The bionic response metrics during the obstacle crossing and recovery step were used for the perturbed walking correlation analysis.

### Gross neuromodulated bionic limb functionality

We assessed overall bionic functionality during locomotion by applying a PCA over the following metrics: peak power, net work, speed, LEK symmetry of level-ground walking at maximum speed and terrain adaptation, stance PO and swing FC for level-ground walking at maximum speed and slope adaptation, stance WA and swing foot placement for stair descent and stance PU and swing FC for stair ascent. To have a consistently positive sign for biomimetic features, the negative peak power and net work signs were reversed for stair descent. We applied a PCA over the following metrics to assess gross bionic functionality during perturbed walking: modified swing kinematics during obstacle crossing, peak power and net work during recovery step, and perturbed walking speed. All metrics were normalized using their mean and standard deviation before applying PCA. We used the PC1 and PC1 score to represent each individual’s overall bionic functionality.

### Statistics

All bionic gait and residual limb muscle neuromechanics results were reported as mean ± s.e.m. Normality of the data was verified by the Shapiro–Wilk test (significance level *α* = 0.05). The effects of the three AMI walking speeds on peak power and net work were analyzed using repeated measures analysis of variance. Sphericity was tested with Mauchly’s sphericity test. Differences between peak power and net work for the three different level-ground walking speeds tested in the AMI cohort were evaluated by two-sided paired *t-*tests with Holm–Šidák correction for multiple comparisons. For other normally distributed data, two-sided paired and unpaired *t*-tests were used for within-group and between-group comparisons, respectively. For data that violated normality, Mann–Whitney *U*-tests were used for between-group comparisons. For the correlation analyses between bionic control metrics and agonist–antagonist muscle afferents, Pearson correlations (*r*), 95% confidence intervals (CI), slopes (*m*) and *P* values were reported. We performed statistical analyses using MATLAB 2020b (MathWorks).

### Reporting summary

Further information on research design is available in the Nature Portfolio Reporting Summary linked to this article.

## Online content

Any methods, additional references, Nature Portfolio reporting summaries, source data, extended data, supplementary information, acknowledgements, peer review information; details of author contributions and competing interests; and statements of data and code availability are available at 10.1038/s41591-024-02994-9.

### Supplementary information

Supplementary Information Detailed descriptions of the control algorithm.

Reporting Summary

Supplementary Video 1 The demonstration of versatile neuroprosthetic gait.

Supplementary Video 2 The demonstration of neuroprosthetic gait responsive to perturbation.

### Source data

Source Data Fig. 1 Statistical source data.

Source Data Fig. 2 Statistical source data.

Source Data Fig. 3 Statistical source data.

Source Data Fig. 4 Statistical source data.

Source Data Fig. 5 Statistical source data.

Source Data Extended Data Fig. 2 Statistical source data.

Source Data Extended Data Fig. 4 Statistical source data.

## Figures and Tables

**Extended Data Table 1:**
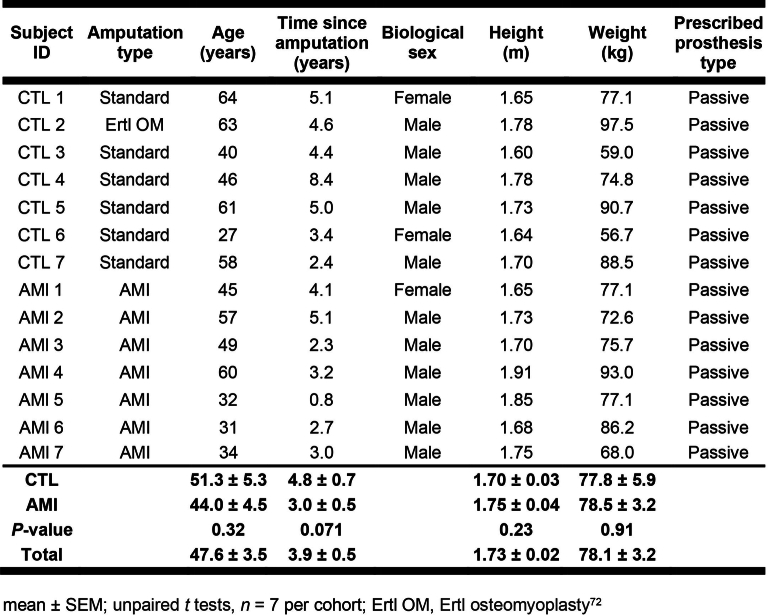
Study population

**Extended Data Table 2:**
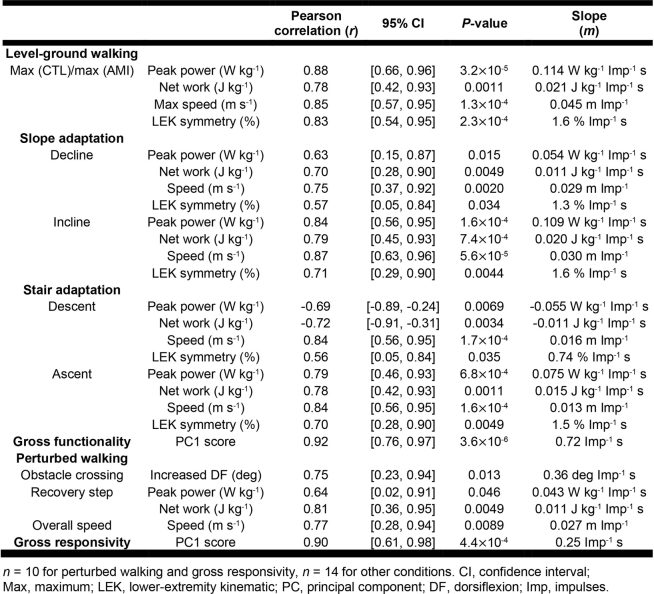
Correlations and slopes between neuromodulated bionic functionality and augmented muscle afferents

## Data Availability

All study data necessary to interpret, verify and extend this work are available in the Supplementary Information. Restrictions apply to the availability of individual participant data that were collected for this study with the informed consent form signed by the research team and study participants in advance of data collection and are, thus, not publicly available. All requests for data should be made to the corresponding author and will be evaluated according to institution policies to determine whether the data requested are subject to any intellectual property or patient privacy obligations. Requests may be made to hherr@media.mit.edu; response time will be within approximately 30 business days. Source data are provided with this paper.

## Code availability

Detailed descriptions of the control algorithm used in this study are provided in the Supplementary Information. Code will be available upon reasonable request to the corresponding author.
