# Supplementary material for: Continuous neural control of a bionic limb restores biomimetic gait after amputation
Source: Nat Med. 2024 Jul 1;30(7):2010–9. doi: 10.1038/s41591-024-02994-9 (PMC11271427; doi:10.1038/s41591-024-02994-9)
Supplement: Supplementary file 1 — Detailed descriptions of the control algorithm. [file 41591_2024_2994_MOESM1_ESM.pdf]

nature medicine

Supplementary information

https://doi.org/10.1038/s41591-024-02994-9

Continuous neural control of a bionic limb restores biomimetic gait after amputation

In the format provided by the authors and unedited

Detailed descriptions of the control algorithm

In this section, we provided detailed descriptions of the control algorithm developed in this study. The aim of the controller was to emulate intact biological ankle behaviors for given muscle activity and the current prosthetic ankle angle and velocity. The controller involved three processes: (i) electromyography (EMG) signal processing, (ii) motor intention decoding, and (iii) emulation of biological ankle angle- and velocity-dependent torque characteristics.

Raw EMG signals from major dorsiflexor (tibialis anterior, TA) and plantar flexor (gastrocnemius, GAS) were recorded at 2kHz and band-passed with a finite impulse response (FIR) filter (stop-band: 0-60 Hz, >360 Hz, pass-band: 90-330 Hz, a stop-band attenuation of 85 dB, order: 198) and cumulative histogram filter[1] to facilitate robust reading within the liner-socket system. Then, root-mean-square (RMS) of rectified EMG (200 ms window size) was normalized by using its minimum and maximum values to compute the EMG envelope. We computed muscle activity (A) from the EMG envelope using bilinear muscle activation dynamics[2] (time constants: t_{act} = 10 ms, t_{deact} = 50 ms).

We decoded motor commands using the TA and GAS muscle activities (A_{TA} and A_{GAS}) by continuously estimating two control variables: the target equilibrium joint angle (θ_{ref}) and impedance modulation level (μ_Z). θ_{ref} was computed by the weighted muscle pair activity differences ($k_{\mathrm{GAS}}A_{\mathrm{GAS}} - k_{\mathrm{TA}}A_{\mathrm{TA}}$) filtered by a critically damped mass-damper-stiffness model (2nd order low-pass filter, cut-off frequency = 6 Hz). We determined the coefficients k_{GAS} and k_{TA} so that θ_{ref} varied between maximum dorsiflexion ($\theta_{\mathrm{max,DF}}$) and plantar flexion ($\theta_{\mathrm{max,PF}}$) of the powered prosthetic ankle. μ_Z was computed by the weighted muscle pair activity sum ($k_{\mathrm{GAS}}A_{\mathrm{GAS}} + k_{\mathrm{TA}}A_{\mathrm{TA}}$) normalized by its maximum value to vary between 0 and 1. Due to differences in motor coordination during dorsiflexion (DF) and plantar flexion (PF), the maximum value of $k_{\mathrm{GAS}}A_{\mathrm{GAS}} + k_{\mathrm{TA}}A_{\mathrm{TA}}$ was different for DF and PF. Therefore, we used different maximum values (N_{DF} and N_{PF}) to normalize $k_{\mathrm{GAS}}A_{\mathrm{GAS}} + k_{\mathrm{TA}}A_{\mathrm{TA}}$ depending on prosthetic actuation direction. The actuation direction was determined by the sign of the difference (δ) between θ_{ref} and the actual prosthetic ankle angle (θ); DF: $\delta < 0$, PF: $\delta \geq 0$. Subject-specific, neural-decoding parameters were determined based on EMG profiles during full DF and PF movements of the phantom ankle joint while each subject stood on their biologically-intact and bionic limbs.

The target joint torque command (τ_{ref}) consisted of active (τ_{act}) and passive (τ_{psv}) torques. We implemented both τ_{act} and τ_{psv} based on the impedance control architecture[3–5], i.e. $\tau_{\mathrm{act}} = K(\theta, \dot{\theta}, A)\delta$, $\tau_{\mathrm{psv}} = K_{\mathrm{psv}}\delta - D_{\mathrm{psv}}\dot{\theta}$. $K(\theta, \dot{\theta}, A)$ was modeled with a biological ankle angle-torque (K_θ) and velocity-torque (K_v) characteristics[6,7] and was scaled by μ_Z, i.e. $K(\theta, \dot{\theta}, A) = K_\theta K_v \mu_Z$. Meanwhile, $|\delta|$ has θ dependent range that can vary as $|\delta| \leq |\delta_{\mathrm{DF}}| = |\theta_{\mathrm{max,DF}} - \theta|$ for DF and $|\delta| \leq |\delta_{\mathrm{PF}}| = |\theta_{\mathrm{max,PF}} - \theta|$ for PF. To cancel out this additional joint angle dependency (note that the angle dependent biological ankle characteristics were accounted for by K_θ), δ was scaled as $\delta/|\delta_{\mathrm{DF}}|$ for DF and $\delta/|\delta_{\mathrm{PF}}|$ for PF. Meanwhile, to avoid the denominators becoming zero, $|\delta_{\mathrm{DF}}|$ and $|\delta_{\mathrm{PF}}|$ were maintained at 1 deg for values that were below 1 deg. This is equivalent to gradually decreasing the maximum joint torque to zero when

the ankle is less than l deg from the end of the powered prosthetic ankle range of motion (ROM) and being driven towards it (software hard-stop). Because the K_θ and K_v are different for DF and PF, we implemented corresponding joint characteristics for each movement (DF: $K_{\theta,\mathrm{DF}}$ and $K_{v,\mathrm{DF}}$, PF: $K_{\theta,\mathrm{PF}}$ and $K_{v,\mathrm{PF}}$) using numerical biological ankle models from previous literature[6,7]. Altogether, $\tau_{\mathrm{act}} = K_{\theta,\mathrm{DF}} K_{v,\mathrm{DF}} \mu_Z \delta / |\delta_{\mathrm{DF}}|$ for DF and $\tau_{\mathrm{act}} = K_{\theta,\mathrm{PF}} K_{v,\mathrm{PF}} \mu_Z \delta / |\delta_{\mathrm{PF}}|$ for PF where DF: $\delta < 0$, PF: $\delta \geq 0$. Notably, for the maximum DF or PF motor intention (i.e. $\mu_Z = 1$, $\delta / |\delta_{\mathrm{DF}}| = -1$ or $\delta / |\delta_{\mathrm{PF}}| = 1$), τ_{act} follows the maximum DF or PF active joint torque values of the biological ankle for given joint angle and velocity (i.e. $-K_{\theta,\mathrm{DF}} K_{v,\mathrm{DF}}$ for DF and $K_{\theta,\mathrm{PF}} K_{v,\mathrm{PF}}$ for PF) unless the ankle is near and being driven towards the end of ROM. For non-maximum DF or PF motor intention, τ_{act} interpolates the active joint torque values based on the impedance control structure (i.e. target joint angle and impedance modulation). The passive joint impedance was set as $K_{\mathrm{psv}} = 0.45$ Nm deg^{-1} and $D_{\mathrm{psv}} = 0.02$ Nm s deg^{-1} to ensure a joint stability under zero muscle activity.

A single board computer (SBC; ODROID-XU4, Hardkernel, South Korea) performed high-level computations for bionic control at 1kHz. The SBC communicated with low-level motor drivers (FlexSEA[8], Dephy, USA) and sensor units using a controller area network (CAN) communication protocol. The joint angle was measured by 14-bit magnetic encoders (AS5047P, ams AG, Austria) placed at the prosthetic joint, and torque values were computed from a prosthetic ankle moment arm and force measurements by strain gauges (MMF307425, Micro-Measurements, USA). Low-level torque control was implemented by back calculation of target force values from target torque values using the moment arm, which was then achieved by proportional control with damping injection based on force feedback from the strain gauges.

Supplementary References

1. Yeon, S. H. & Herr, H. M. Rejecting impulse artifacts from surface EMG signals using real-time cumulative histogram filtering. *IEEE Int. Conf. Eng. Med. Biol. Soc.* 6235–6241 (2021) doi:10.1109/EMBC46164.2021.9631052.
2. Markowitz, J. *et al.* Speed adaptation in a powered transtibial prosthesis controlled with a neuromuscular model. *Phil. Trans. R. Soc. B* **366**, 1621–1631 (2011).
3. Hogan, N. Adaptive control of mechanical impedance by coactivation of antagonist muscles. *IEEE Trans. Automat. Contr.* **29**, 681–690 (1984).
4. Hogan, N. The mechanics of multi-joint posture and movement control. *Biol. Cybern.* **52**, 315–331 (1985).
5. Lee, H. & Hogan, N. Time-varying ankle mechanical impedance during human locomotion. *IEEE Trans. Neural Syst. Rehabil. Eng.* **23**, 755–764 (2015).
6. Hasson, C. J., Miller, R. H. & Caldwell, G. E. Contractile and elastic ankle joint muscular properties in young and older Adults. *PLOS ONE* **6**, e15953 (2011).
7. Anderson, D. E., Madigan, M. L. & Nussbaum, M. A. Maximum voluntary joint torque as a function of joint angle and angular velocity: Model development and application to the lower limb. *J. Biomech.* **40**, 3105–3113 (2007).
8. Duval, J.-F. & Herr, H. M. FlexSEA: Flexible, scalable electronics architecture for wearable robotic applications. *IEEE Int. Conf. Biomed. Robot. Biomechatronics* 1236–1241 (2016) doi:10.1109/BIOROB.2016.7523800.
