# Supplementary material for: Continuous neural control of a bionic limb restores biomimetic gait after amputation
Source: Nat Med. 2024 Jul 1;30(7):2010–9. doi: 10.1038/s41591-024-02994-9 (PMC11271427; doi:10.1038/s41591-024-02994-9)
Supplement: Supplementary file 2 — Reporting Summary [file 41591_2024_2994_MOESM2_ESM.pdf]

nature portfolio

Corresponding author(s): Hugh Herr

Last updated by author(s): Apr 1, 2024

Reporting Summary

Nature Portfolio wishes to improve the reproducibility of the work that we publish. This form provides structure for consistency and transparency in reporting. For further information on Nature Portfolio policies, see our Editorial Policies and the Editorial Policy Checklist.

Statistics

For all statistical analyses, confirm that the following items are present in the figure legend, table legend, main text, or Methods section.

n/a	Confirmed	
☐	☒	The exact sample size (n) for each experimental group/condition, given as a discrete number and unit of measurement
☐	☒	A statement on whether measurements were taken from distinct samples or whether the same sample was measured repeatedly
☐	☒	The statistical test(s) used AND whether they are one- or two-sided<br>*Only common tests should be described solely by name; describe more complex techniques in the Methods section.*
☐	☒	A description of all covariates tested
☐	☒	A description of any assumptions or corrections, such as tests of normality and adjustment for multiple comparisons
☐	☒	A full description of the statistical parameters including central tendency (e.g. means) or other basic estimates (e.g. regression coefficient) AND variation (e.g. standard deviation) or associated estimates of uncertainty (e.g. confidence intervals)
☐	☒	For null hypothesis testing, the test statistic (e.g. F, t, r) with confidence intervals, effect sizes, degrees of freedom and P value noted<br>*Give P values as exact values whenever suitable.*
☒	☐	For Bayesian analysis, information on the choice of priors and Markov chain Monte Carlo settings
☒	☐	For hierarchical and complex designs, identification of the appropriate level for tests and full reporting of outcomes
☐	☒	Estimates of effect sizes (e.g. Cohen's d, Pearson's r), indicating how they were calculated

Our web collection on statistics for biologists contains articles on many of the points above.

Software and code

Policy information about availability of computer code

Data collection	EMG data were collected using a portable EMG sensor unit and flexible bi-polar electrodes previously developed in our group (S. H. Yeon et al, 2021). The algorithm used for bionic testing is available in Supplementary Information. Code will be available upon reasonable request to the corresponding author. Lower-extremity kinematic data were collected using a Wireless Twin-axis Goniometer (Biometrics ltd, UK). Fascicle strain was recorded using a high-definition real-time ultrasound scanner (LS128, Telemed, Lithuania).
Data analysis	We performed all statistical analyses using MATLAB 2020b (Mathworks, USA). The algorithm used for bionic testing is available in Supplementary Information. The fascicle strain was estimated from ultrasound video recordings using the Ultra Track V2 MATLAB package.

For manuscripts utilizing custom algorithms or software that are central to the research but not yet described in published literature, software must be made available to editors and reviewers. We strongly encourage code deposition in a community repository (e.g. GitHub). See the Nature Portfolio guidelines for submitting code & software for further information.

Data

Policy information about availability of data

All manuscripts must include a data availability statement. This statement should provide the following information, where applicable:

- Accession codes, unique identifiers, or web links for publicly available datasets
- A description of any restrictions on data availability
- For clinical datasets or third party data, please ensure that the statement adheres to our policy

All study data necessary to interpret, verify, and extend this work are available in the Supplementary Information. Restrictions apply to the availability of individual participant data that were collected for this study with the informed consent form signed by the research team and study participants in advance of data collection and are, thus, not publicly available. All requests for data should be made to the corresponding author and will be evaluated according to institution policies to determine whether the data requested are subject to any intellectual property or patient privacy obligations. Requests may be made to hherr@media.mit.edu; response time will be within approximately 30 business days.

Research involving human participants, their data, or biological material

Policy information about studies with human participants or human data. See also policy information about sex, gender (identity/presentation), and sexual orientation and race, ethnicity and racism.

Reporting on sex and gender	The study involved 11 males and 3 females. Sex was self-reported. The gender information (shaped by social and cultural circumstances) was not collected. The study was designed to assess the impact of the AMI amputation procedure in persons with transtibial amputation compared to those who underwent a non-AMI amputation procedure (CTL). Thus, neither sex nor gender were considered in this study.
Reporting on race, ethnicity, or other socially relevant groupings	We did not collect or use any covariates regarding race, ethnicity, and other social groupings at any stage of the study.
Population characteristics	Fourteen individuals with unilateral transtibial amputations participated in the study. Seven of these subjects had undergone an Agonist-antagonist Myoneural Interface (AMI) amputation (AMI cohort) and seven subjects had undergone non-AMI amputation (CTL cohort). The matching criteria between AMI and CTL subjects were subject age, time since amputation, height, and weight. Subjects had following characteristics (mean ± SEM): age = 47.6 ± 3.5 years; time since amputation = 3.9 ± 0.5 years; height = 1.73 ± 0.02 m; weight = 78.1 ± 3.2.
Recruitment	AMI group subjects were drawn from the pool of patients who had undergone an AMI amputation procedure at Brigham and Women's Hospital (BWH) in Boston MA under Partners Healthcare IRB protocol P2014001379. CTL group subjects were recruited by word of mouth or external inquiries. Potential subjects spoke to a team member who described the study, answered questions, and provided links to the Clinicaltrials.gov website (NCT 03913273). Individuals who decided to participate signed informed consent form at Massachusetts Institute of Technology (MIT) in Cambridge MA. Study data were collected under approval of MIT's Committee on the Use of Humans as Experimental Subjects (protocol 1812634918). The study protocol can be made available upon reasonable request to the corresponding authors. The participants were compensated on an hourly basis for the study (USD 20 per hour). All subjects met the following inclusion criteria: age within the range of 18 years to 65 years; AMI transtibial amputation (AMI group) or Non-AMI transtibial amputation (CTL group); fully healed amputation site; proficiency in using a standard lower extremity prosthesis; activity or K-Level of at least K3 to K4 (capability to ambulate with variable cadence). There were no appreciable self-selection bias or other biases that affected recruitment.
Ethics oversight	The study protocol was approved by the MIT Committee on the Use of Humans as Experimental Subjects, which is MIT's IRB.

Note that full information on the approval of the study protocol must also be provided in the manuscript.

Field-specific reporting

Please select the one below that is the best fit for your research. If you are not sure, read the appropriate sections before making your selection.

☒ Life sciences ☐ Behavioural & social sciences ☐ Ecological, evolutionary & environmental sciences

For a reference copy of the document with all sections, see nature.com/documents/nr-reporting-summary-flat.pdf

Life sciences study design

All studies must disclose on these points even when the disclosure is negative.

Sample size	Sample size was based on and the same as the size of our free-space motor control dataset published in Commun. Med. (H. Song et al., 2022).
Data exclusions	No data were excluded from the analyses.
Replication	All bionic gait experiments successfully showed consistent results in fourteen independent subjects with below-knee amputations.

Randomization	The study was designed to assess the impact of the AMI amputation procedure in persons with transtibial amputation compared to those who underwent a non-AMI amputation procedure (CTL). Thus, participants in each cohort were determined by the type of amputation procedure they underwent (CTL or AMI). All fourteen participants (7 CTL, 7 AMI) underwent the same bionic gait testing protocol. To help ensure participants' safety, bionic gait testing was performed in an order of increasing degree of difficulty proceeding from level-ground walking to navigating slopes and stepping stairs. For the additional exploratory perturbed walking trial, ten participants (6 CTL, 4 AMI) participated based on their availability.
Blinding	Due to the nature of the study design, blinding for the subjects' tested conditions (CTL or AMI) was not feasible. However, the analyses were conducted in both group comparisons and subject-specific features.

Reporting for specific materials, systems and methods

We require information from authors about some types of materials, experimental systems and methods used in many studies. Here, indicate whether each material, system or method listed is relevant to your study. If you are not sure if a list item applies to your research, read the appropriate section before selecting a response.

Materials & experimental systems

n/a	Involved in the study
☒	☐ Antibodies
☒	☐ Eukaryotic cell lines
☒	☐ Palaeontology and archaeology
☒	☐ Animals and other organisms
☐	☒ Clinical data
☒	☐ Dual use research of concern
☒	☐ Plants

Methods

n/a	Involved in the study
☒	☐ ChIP-seq
☒	☐ Flow cytometry
☒	☐ MRI-based neuroimaging

Clinical data

Policy information about clinical studies

All manuscripts should comply with the ICMJE guidelines for publication of clinical research and a completed CONSORT checklist must be included with all submissions.

Clinical trial registration	The ClinicalTrials.gov Registration for this work is NCT03913273.
Study protocol	Information about the clinical trial study protocol NCT03913273, which has as a secondary ID R01HD097135, is publicly accessible at https://clinicaltrials.gov/ct2/show/NCT03913273. The study record was first posted in April 2019 and is regularly updated, most recently in July 2023.
Data collection	All data were collected at the Massachusetts Institute of Technology. The period of recruitment and data collection was from April 2019 to January 2023.
Outcomes	To address the clinical trial aim of determining whether AMIs can improve prosthetic terrain adaptations, we assessed two pre-defined outcome measures: swing phase plantar flexion (PF) control during stair descent and swing phase dorsiflexion (DF) control during stair ascent.

Plants

Seed stocks	n/a
Novel plant genotypes	n/a
Authentication	n/a
